# Enzymatically synthesized glycogen prevents ultraviolet B-induced cell damage in normal human epidermal keratinocytes

**DOI:** 10.3164/jcbn.20-44

**Published:** 2020-06-05

**Authors:** Yasukiyo Yoshioka, Tomoya Kitakaze, Takakazu Mitani, Takashi Furuyashiki, Hitoshi Ashida

**Affiliations:** 1 Department of Clinical Nutrition and Dietetics, Faculty of Clinical Nutrition and Dietetics, Konan Women’s University, 6-2-23 Morikita-machi, Higashinada-ku, Kobe 658-0001, Japan; 2 Graduate School of Science, Technology and Innovation, Kobe University, 1-1 Rokkodai-cho, Nada-ku, Kobe 651-8501, Japan; 3 Department of Agrobioscience, Graduate School of Agricultural Science, Kobe University, 1-1 Rokkodai-cho, Nada-ku, Kobe 651-8501, Japan; 4 Institute of Health Sciences, Ezaki Glico Co., Ltd., 4-6-5 Utajima, Nishiyodogawa-ku, Osaka 555-8502, Japan

**Keywords:** enzymatically synthesized glycogen, ultraviolet B, reactive oxygen species, anti-oxidative protein, normal human epidermal keratinocytes

## Abstract

Enzymatically synthesized glycogen is a product from starch. Enzymatically synthesized glycogen has been reported to possess various health beneficial effects such as anti-oxidative and anti-inflammatory effects. In this study, we investigated the effect of enzymatically synthesized glycogen on ultraviolet B-induced oxidative stress and apoptosis in normal human epidermal keratinocytes. Treatment with enzymatically synthesized glycogen suppressed ultraviolet B-induced reactive oxygen species, caspase-3 activity, and DNA fragmentation in normal human epidermal keratinocytes. Furthermore, enzymatically synthesized glycogen increased in the expression level of heme oxygenase-1, NAD(P)H: quinone oxidoreductase 1, and NF-E2-related factor 2, a transcriptional factor for heme oxygenase-1 and NAD(P)H: quinone oxidoreductase 1. Although enzymatically synthesized glycogen did not increase in its mRNA expression level of NF-E2-related factor 2, enzymatically synthesized glycogen retained its protein degradation. Knockdown of heme oxygenase-1 and NAD(P)H: quinone oxidoreductase 1 canceled enzymatically synthesized glycogen-suppressed reactive oxygen species accumulation in normal human epidermal keratinocytes. It is, therefore, concluded that enzymatically synthesized glycogen inhibited ultraviolet B-induced oxidative stress through increasing the expression level of heme oxygenase-1 and NAD(P)H: quinone oxidoreductase 1 through the NF-E2-related factor 2 pathway in normal human epidermal keratinocytes.

## Introduction

Skin is the largest gross area organ of the human body and protects the organism against exogenous physical, chemical, and biological damages, including ultraviolet (UV) radiation. The chronic exposure to solar UVB radiation (280–320 nm) is one of the most important factors for development of various skin diseases. External exposure of UV radiation has biological stresses, such as sunburn, hyperpigmentation, solar keratosis, and solar elastosis.^(1)^ It has known that UVB irradiation increases the level of reactive oxygen species (ROS), which results in oxidative damage of cellular substrates, leading to inflammation, apoptosis, immunosuppression, and gene mutation.^(2)^ UVB radiation has been shown to induce IL-10, IL-4, IL-6, and TNF-α.^(3–5)^ Both acute and chronic UVB exposure has been associated with immunosuppression that is to reduce the risk of harmful and excessive inflammation in the skin.^(6–8)^ UVB radiation-induced immunosuppression occurs via down-regulation of T-cell mediated immunity through the induction of immune-modulating cytokines.^(9)^ Furthermore, it is reported that methylparaben, which is used as a preservative in cosmetics, may have harmful effects on human skin when exposed to sunlight.^(10)^ It is, therefore, important to investigate safe and effective care, such as medical creams and cosmetics, to protect UVB irradiation.

The increased production of ROS leads to the oxidation and damage to cellular molecules, resulting in the altered functions.^(11)^ In response to excessive amounts of ROS, a variety of transcription factors are activated including NF-E2-related factor 2 (Nrf2), nuclear factor kappa B (NF-κB), and activator protein 1. The transcription factor, Nrf2 is a major transactivator of cytoprotective genes in response to oxidative stress and xenobiotic electrophiles. Nrf2 regulates the transcription of cytoprotective genes after binding to cis-acting elements.^(12)^ The antioxidant response elements present in the enhancer regions of these genes.^(13)^

Enzymatically synthesized glycogen (ESG) has been produced from plant starch.^(14)^ The physical properties of ESG and glycogen from natural sources were similar, whereas molecular structures of these polysaccharides were slightly different: α-1,6 Linkages in the ESG are located in the center of the molecule, and those of natural glycogens are located near the surface of the molecule.^(15)^ Therefore, ESG is partially digested with α-amylase, and the indigestive ESG called resistant glycogen (RG).^(16)^ Recently, RG has reported to have antioxidant activity, resulting in suppression of colitis in mice.^(17)^ Furthermore, ESG has reported to protect urban particulate matter-induced inflammation in normal human epidermal keratinocytes (NHEK).^(18)^ However, the effect of ESG on UVB-induced cellular damages remains unclear yet. In this study, we investigated whether ESG suppressed UVB-induced ROS accumulation and apoptosis in NHEK. We further investigated whether ESG increased heme oxygenase-1 (HO-1) and NAD(P)H: quinone oxidoreductase 1 (NQO1) expressions through Nrf2 pathway.

## Materials and Methods

### Reagents

ESG was prepared from plant starch using three enzymes as previously described.^(19)^ The molecular weight of ESG is approximately 8,700 kDa. RG was prepared by partial digestion of ESG with α-amylase.^(20)^ Oyster glycogen (OG) was purchased from FUJIFILM Wako Pure Chemical Co. (Osaka, Japan). 2',7'-Dichlorodohydrofluorescein diacetate (DCFH-DA) and 4',6-diamino-2-phenylindole (DAPI) was purchased from Sigma Aldrich (St. Louis, MO). Antibody against HO-1 was Enzo Life Sciences (Lausen, Switzerland). Antibodies against NQO1, Nrf2, caspase-3, -8, and -9 were from Santa Cruz Biotechnology (Santa Cruz, TX). Antibodies against cleaved capase-3 and β-actin were from Cell Signaling Technology (Denver, MA). Antibody against Ser phosphate was from Abcam (Cambridge, United Kingdom). Horseradish peroxidase (HRP)-conjugated anti-rabbit IgG was from Bio-Rad Laboratories Inc. (Hercules, CA). Blocking One solution was from Nacalai Tesque, Inc. (Kyoto, Japan). Polyvinylidene difluoride membrane was from GE Healthcare (Fairfield, WA). ImmunoStar LD chemiluminescence detection kit was a product of FUJIFILM Wako Pure Chemical Co. All other reagents were of the highest grade commercially available.

### Cell culture and UVB irradiation

NHEK was purchased from KURABO industries (Osaka, Japan) and cultured with a complete medium, EpiLife (Thermo Fisher Scientific, Waltham, MS) supplemented with the cell growth addition agents [10 µg/ml insulin, 0.1 ng/ml human recombinant epidermal growth factor, 0.67 µg/ml hydrocortisone hemisuccinate, 50 µg/ml gentamycin, 50 ng/ml amphotericin B, and 0.4% (v/v) bovine pituitary gland body extracts] at 37°C under an atmosphere of 5% CO_2_ in air. ESG, RG, and OG were dissolved in distilled water and diluted with the medium to appropriate concentrations. The final volume of vehicle was adjusted to 0.1% (v/v). For UVB irradiation, NHEK was irradiated with UVB (312 nm, 20 mJ/cm^2^) using an UVB generator (BioDoc-It System, UVP Ltd., Upland, CA). To minimize absorption of the radiation by the medium, a thin layer of phosphate-buffered saline was left above the cells during UVB exposure.

### Measurement of ROS accumulation

Accumulation of intracellular ROS was determined using DCFH-DA. NHEK was incubated with polysaccharides for 24 h. After washing with PBS, the cells were exposed by UVB irradiation (20 mJ/cm^2^), followed by incubation with 20 µM DCFH-DA and 1 µg/ml DAPI for 30 min. The fluorescence of DCF and DAPI was measured at 485/535 nm and 355 and 460 nm, respectively, using a plate reader (ALVO, PerkinElmer Inc, Wellesley, MA). The fluorescence intensity of DCF was normalized by that of DAPI.

### Western blotting

NHEK was incubated with several concentrations of polysaccharides for 24 h. For the degradation assay using cycloheximide as an inhibitor of protein synthesis, NHEK was incubated with 10 µg/ml cycloheximide in the presence or absence of 600 µg/ml polysaccharides for 0, 30, 60, 120 and 180 min. The cells were lysed with a lysis buffer, pH 7.5, containing 50 mM Tris, 150 mM NaCl, 0.5% (w/v) Nonidet P-40, 10 mM sodium pyrophosphate, 2 mM EDTA, 1 mM phenylmethylsulfonyl fluoride, and 10 µg/ml leupeptin. The lysate was centrifuged at 20,000 × *g* for 20 min and the supernatant was used as the cell lysate. The cell lysate was mixed with sodium dodecyl sulfate sample buffer consisting of 62.5 mM Tris, pH 6.8, 2% (w/v) sodium dodecyl sulfate, 10% (v/v) glycerol, 5% (v/v) 2-mercaptoethanol, and 0.02% (w/v) bromophenol blue. The mixture was incubated at 100°C for 5 min and subjected to sodium dodecyl sulfate-polyacrylamide gel electrophoresis. Separated proteins in the gel were transferred onto a polyvinylidene fluoride membrane. The membrane was incubated with a blocking solution consisting of Blocking One for 1 h at room temperature and treated with primary antibodies overnight at 4°C, followed by the corresponding horseradish peroxidase-conjugated secondary antibody for another 1 h at room temperature. Protein bands were visualized using ImmunoStar LD Western Blotting Substrate and detected with Light-Capture II (ATTO, Tokyo, Japan). The density of specific band was determined using ImageJ image analysis software (National Institutes of Health, Bethesda, MD).

### RNA interference

The target sequences for HO-1, NQO1, and negative control siRNA duplexes were as follows: siHO-1, 5'-CAAAUGCAGUAUUUUUGUUTT-3'; siNQO1, 5'-AGCCU UUCAGAAUGGCUGGCTT-3' and siCont, 5'-AAGUAACAC UUGGCUAUUUCUTT-3'. These duplexes were introduced into NHEK using Lipofectamine^®^ RNAiMAX reagent (Thermo Fisher Scientific) for 48 h according to the manufacturer’s instructions.

### RNA isolation and quantitative real-time PCR

Total RNA from NHEK was isolated using TRIzol (Thermo Fisher Scientific) in accordance with the manufacturer’s instructions, and subjected to the reverse-transcriptional reaction. Resultant cDNA was subjected to quantitative real-time PCR using the SYBR PremixEx Taq II (Takara Bio, Kyoto, Japan) and a two-step PCR method on a Thermal Cycler Dice real-time system (Takara Bio). The following specific primers were used: *NFE2L2* (forward primer 5'-GACGGTATGCAACAGGACATTGAG-3' and reverse primer 5'-AACTTCTGTCAGTTTGGCTTCTGGA-3'); and *ACTB* (forward primer 5'-GGACTTCGAGCAAGAGATGG-3' and reverse primer 5'-AGCACTGTGTTGGCGTACAG-3'). *ACTB* mRNA was used as a normalized control.

### Immunoprecipitation

NHEK was cultured with 600 µg/ml polysaccharides for 24 h and lysed with the lysis buffer. The cell lysate was incubated with anti-Nrf2 antibody (Santa Cruz) overnight and then treated with protein G-Sepharose resin (GE Helthcare, Milwaukee, MI) for 2 h at 4°C. After washing with the lysis buffer, the complex was subjected to Western blotting.

### Measurement of caspases-3, -8, and -9 activities

 NHEK was cultured with several concentrations of polysaccharides for 24 h and lysed. For the enzymatic activity assay, the cell lysate was incubated with each peptide substrate corresponding caspases-3, -8 and -9 for 1 h at 37°C. The tetrapeptide fluorogenic substrates for caspases-3, -8 and -9 were Ac-DEVD-7-amino-4-methyl-coumarin (AMC), Ac-IETD-AMC and Ac-LEHD-AMC, respectively. After the reaction, the fluorescence intensity of AMC was measured at 380/460 nm using the plate reader. For detection of cleaved caspase proteins, the cell lysate was subjected to Western blotting.

### DNA fragmentation assay

For extraction of DNA, NHEK was incubated with several concentrations of polysaccharides for 24 h and lysed. After the cell lysate was centrifuged at 3,500 rpm for 5 min. The supernatant was added 10% (w/v) sodium dodecyl sulfate and incubated for another 2 h at 50°C. The mixture was added 3M sodium acetate, 1 µg/ml glycogen and ethanol, and then centrifuged at 15,000 rpm for 5 min. Resultant pellet was suspended in 70% ethanol and centrifuged again at 15,000 rpm for 5 min. Dried pellet was resolved TE buffer and subject to 2% agarose gel electrophoresis. After staining with ethidium bromide, DNA fragmentation was detected using trans illuminator (UVP Ltd.).

### MTT assay

NHEK was incubated with several concentrations of polysaccharides for 24 h and treated with 500 µg/ml MTT reagent for 4 h. After removing the supernatant, resultant pellet was washed with PBS twice and then lysed with 2-propanol containing with 0.04 M hydrogen chloride. Formed formazan was measured at 570 nm using the plate reader.

### Statistical analysis

Statistical analysis was performed with JMP statistical software ver. 11.2.0 (SAS Institute, Cray, NC). Data are represented as the mean ± SD from at least three independent determinations for each experiment. The statistical significance of experimental observations was determined using Tukey-Kramer test. The level of significance was set as *p*<0.05.

## Results

### ESG inhibited UVB-induced ROS accumulation in NHEK

**Fig. 1:**
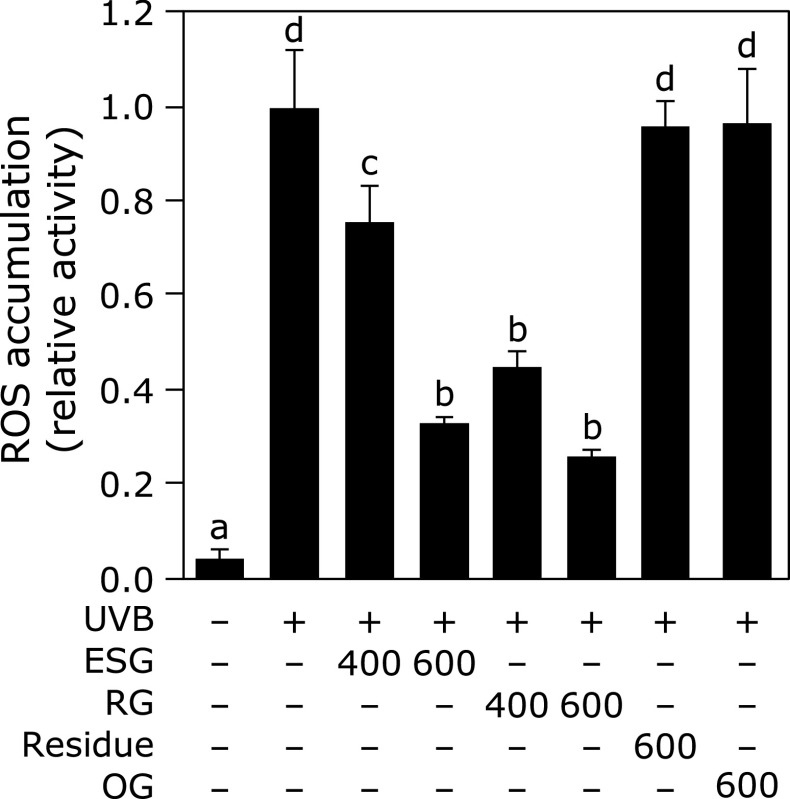
ESG and RG inhibited UVB-induced ROS accumulation in NHEK. UVB-induced ROS accumulation was measured with or without ESG, RG, the residue, and OG in NHEK. NHEK was treated with ESG, RG, the residue, and OG for 24 h. The cells were exposed by UVB irradiation (20 mJ/cm^2^), followed by incubation with 20 µM DCFH-DA and 1 µg/ml DAPI for 30 min. The fluorescence intensity of DCF and DAPI was measured at 485/535 nm and 355 and 460 nm, respectively. The fluorescence intensity of DCF was normalized by that of DAPI. Data are presented as the means ± SD (*n* = 4). Different letters indicate significant differences (*p*<0.05; Tukey-Kramer test).

First, it was examined whether ESG inhibited UVB-induced oxidative stress in NHEK. UVB irradiation increased ROS accumulation in NHEK as expected. ESG and its metabolite RG, but not the residue, which is completely degraded ESG by α-amylase to glucose and oligosaccharides, inhibited UVB-induced ROS accumulation in a dose dependent manner (Fig. 1). Furthermore, OG, a natural source glycogen, had no effect on UVB-induced ROS accumulation (Fig. 1).

### ESG increased the expression of HO-1 and NQO1

**Fig. 2:**
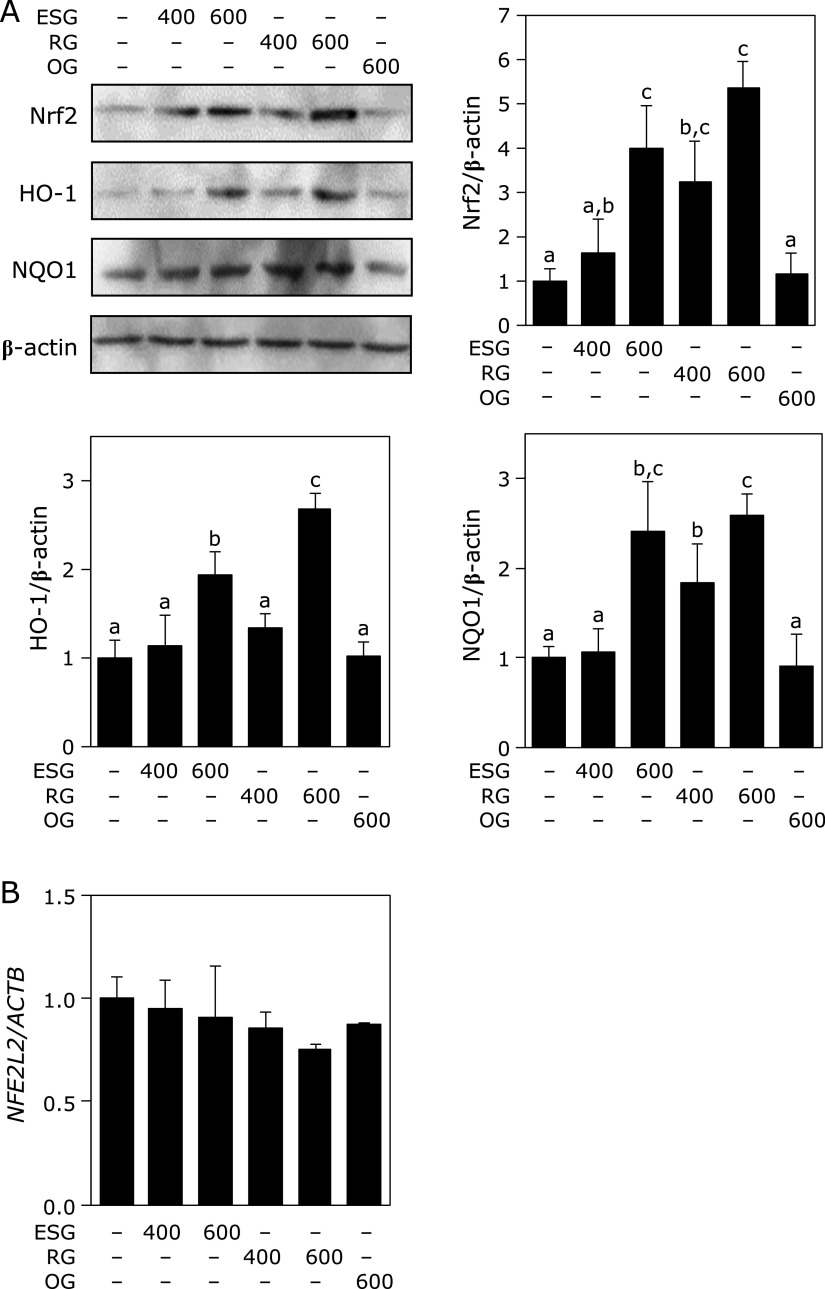
ESG and RG increased in the protein expression level of HO-1, NQO1, and Nrf2 in NHEK. (A) NHEK was treated with ESG, RG, and OG for 24 h and subject to Western blotting for detection of HO-1, NQO1, and Nrf2. (B) NHEK was treated with ESG, RG, and OG for 24 h and subjected to real time PCR for detection of Nrf2 gene (*NFE2L2*). Data are presented as the means ± SD (*n* = 4). Different letters indicate significant differences (*p*<0.05; Tukey-Kramer test).

 To clarify the effect of ESG on the expression level of antioxidant proteins, the expression level of HO-1 and NQO1 and their transcription factor Nrf2 was measured in NHEK. Treatment with ESG or RG, but not OG, increased protein expression level of HO-1, NQO1 and Nrf2 (Fig. 2A). On the contrary, treatment with ESG and RG did not affect expression of Nrf2 gene (*NFE2L2*) (Fig. 2B).

### ESG retained the protein degradation of Nrf2

**Fig. 3:**
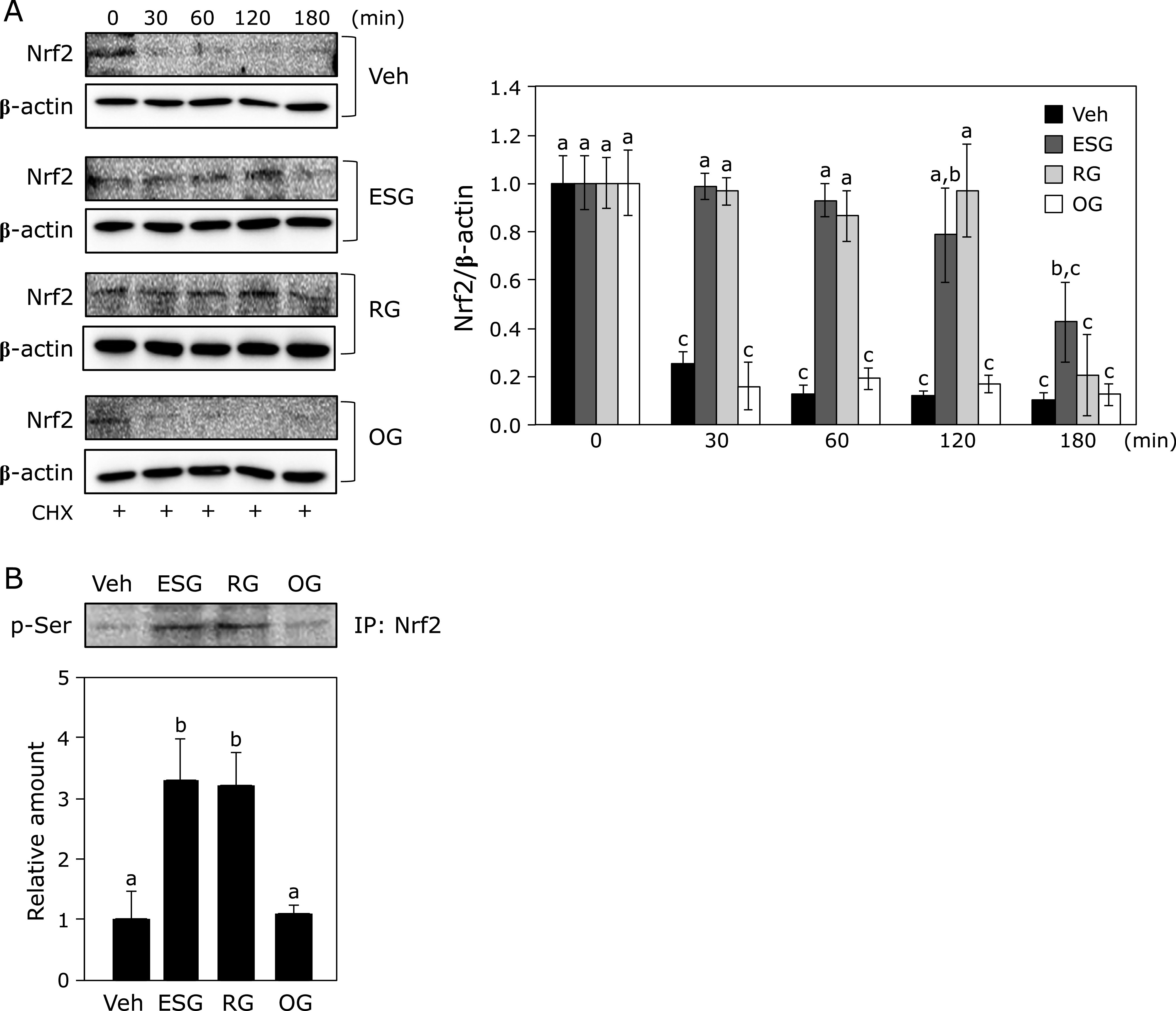
Effect of ESG and RG on Nrf2 stability in NHEK. (A) For time-course analysis of Nrf2 protein expression, NHEK treated with 600 µg/ml ESG, RG, and OG in the presence of 10 µg/ml cycloheximide (CHX) for indicated periods and subject to Western blotting for detection of Nrf2. (B) For detection of serine phosphorylation of Nrf2, Nrf2 protein was immunoprecipitated (IP) in NHEK treated with 600 µg/ml ESG, RG, and OG for 1 h, followed by Western blot analysis using anti-pSer antibody. Data are presented as the means ± SD (*n* = 4). Different letters indicate significant differences (*p*<0.05; Tukey-Kramer test).

To elucidate the mechanism of ESG on the protein expression of Nrf2, the Nrf2 level was monitored in the presence of cycloheximide in NHEK. Nrf2 was degraded in time-dependent manner by treatment with cycloheximide. ESG and RG inhibited Nrf2 degradation in NHEK (Fig. 3A). Furthermore, treatment with ESG and RG promoted phosphorylation of Nrf2 at serine residues (Fig. 3B).

### Involvement of HO-1 and NQO1 in the inhibition of UVB-induced ROS accumulation by ESG

**Fig. 4:**
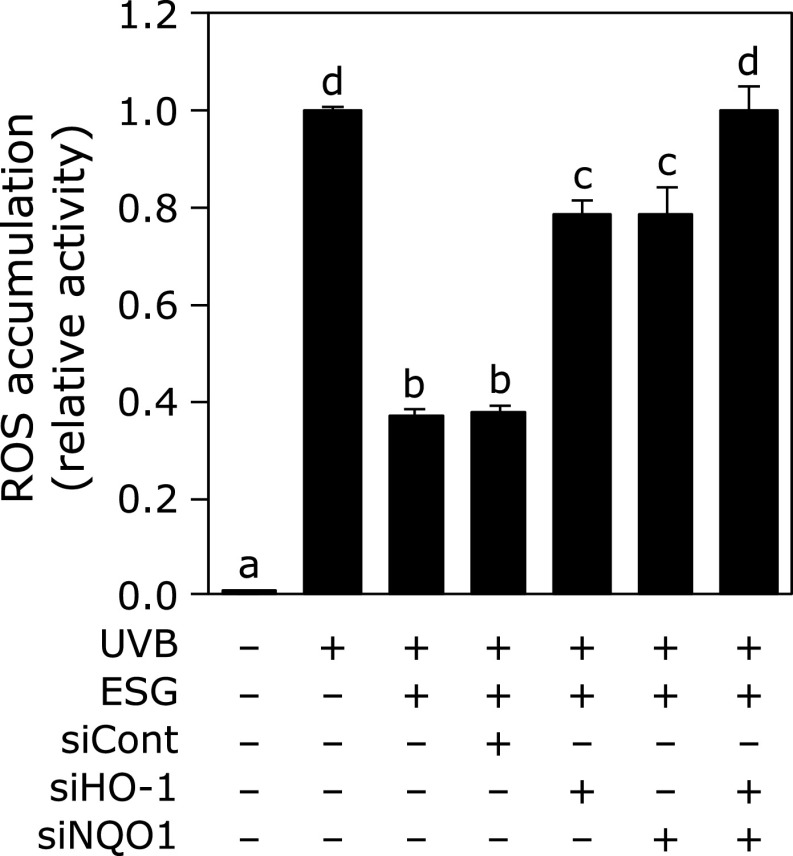
Knockdown of HO-1 and NQO-1 canceled the function of ESG against UVB-induced ROS accumulation in NHEK UVB-induced ROS accumulation was measured in HO-1- and/or NQO1-knocked NHEK as the same procedure as described in Fig. 1. Data are presented as the means ± SD (*n* = 4). Different letters indicate significant differences (*p*<0.05; Tukey-Kramer test).

To clarify the involvement of HO-1 and NQO1 in the inhibitory effect of ESG against UVB-induced oxidative stress, it was investigated the effect of ESG on ROS accumulation in HO-1 and/or NQO1-knockdowned NHEK. ESG partially inhibited UVB-induced ROS accumulation in HO-1 or NQO1-knockdowned NHEK. Furthermore, knockdown of both HO-1 and NQO1 completely canceled ESG-caused antioxidant effect on UVB-induced ROS accumulation (Fig. 4).

### ESG inhibited UVB-induced caspases-3 and -9 enzymatic activity and their activation

**Fig. 5:**
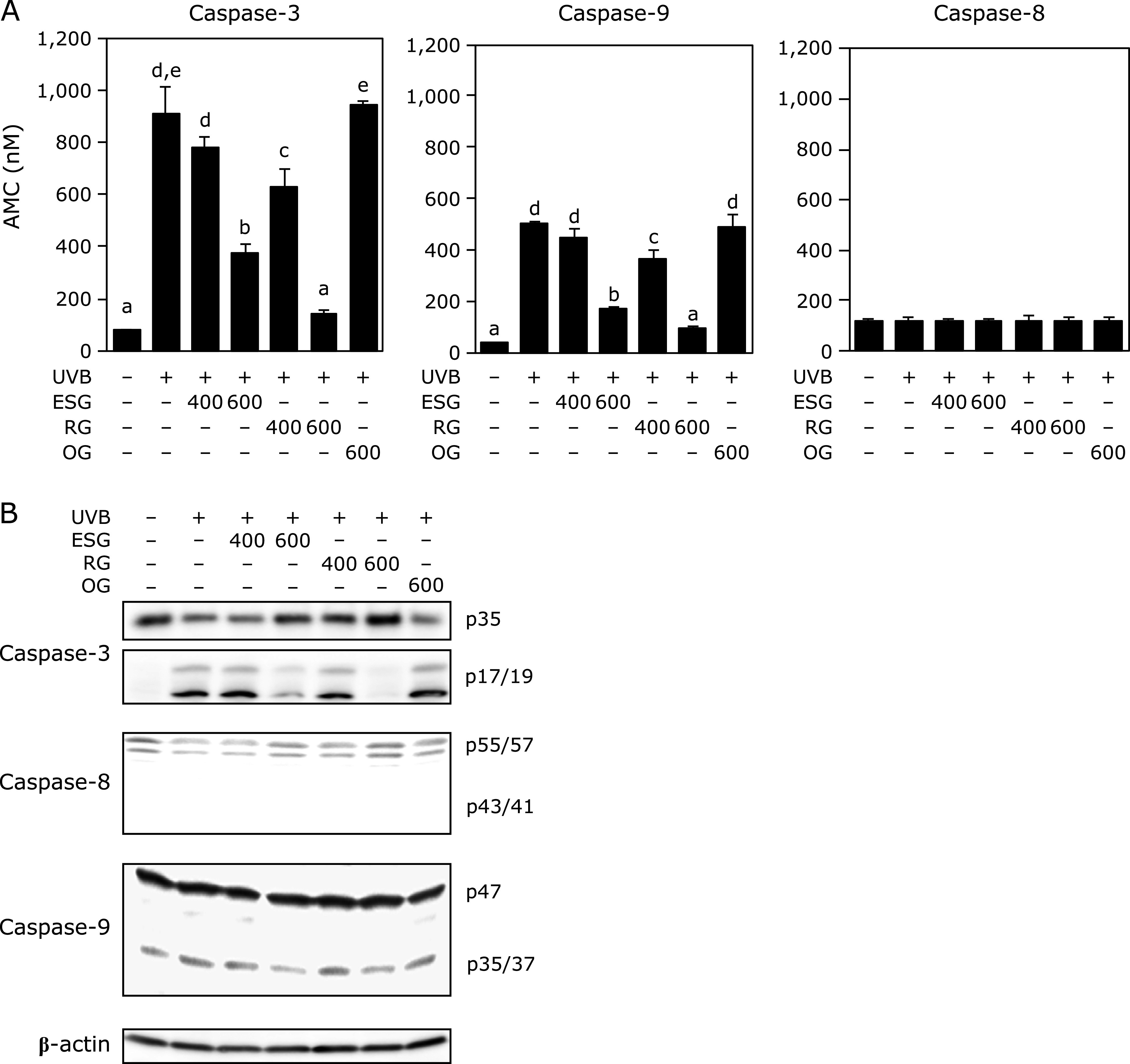
ESG and RG inhibited UVB-induced caspases activity and cleavage in NHEK. UVB-induced apoptosis was evaluated with or without ESG, RG, and OG in NHEK. NHEK was treated with ESG, RG, and OG for 24 h and irradiated with UVB (20 mJ/cm^2^). (A) The activity of caspase-3, -8 and -9 was measured using corresponding peptide substrate by monitoring the released fluorescence intensity of AMC at 380/460 nm in cell lysate. (B) The cleavages of caspase proteins were detected in cell lysate by Western blotting. Data are presented as the means ± SD (*n* = 4). Different letters indicate significant differences (*p*<0.05; Tukey-Kramer test).

Next, we examined the effect of ESG on UVB-induced apoptosis in NHEK. Using the fluorescent peptide substrates, UVB irradiation increased the activity of caspase-3 and -9, accompanied by their protein cleavage in NHEK (Fig. 5). UVB did not activate caspase-8. ESG and RG, but not OG, inhibited UVB-increased caspases-3 and -9 activities (Fig. 5A) and their protein cleavage (Fig. 5B).

### ESG inhibited UVB-induced cytotoxicity and DNA fragmentation

**Fig. 6:**
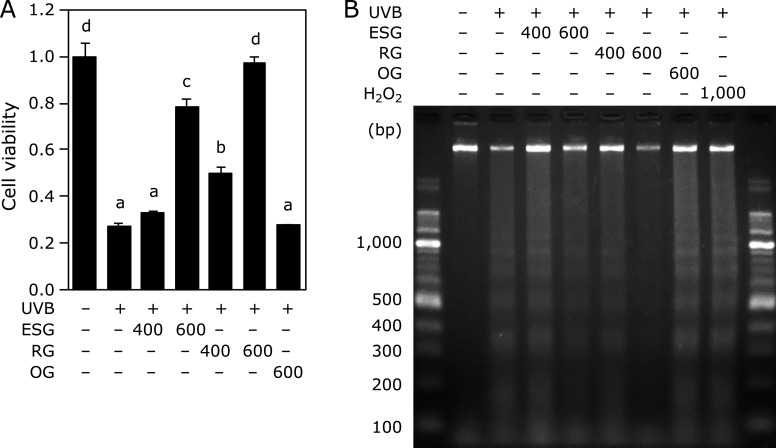
ESG and RG inhibited UVB-induced cytotoxicity and DNA fragmentation in NHEK. NHEK was treated with ESG, RG, and OG for 24 h and irradiated with UVB (20 mJ/cm^2^). (A) Cytotoxicity was measurement by MTT assay. (B) After DNA was extracted, DNA fragmentation was measured by agarose gel electrophoresis. Data are presented as the means ± SD (*n* = 4). Different letters indicate significant differences (*p*<0.05; Tukey-Kramer test).

To further clarify the prevention effect of ESG on UVB-induced apoptosis, cytotoxicity and DNA fragmentation were measured in NHEK. UVB irradiation significantly decreased the cell viability (Fig. 6A) and caused DNA fragmentation (Fig. 6B). ESG and RG, but not OG, inhibited UVB-caused cytotoxicity dose-dependently (Fig. 6A). Furthermore, ESG and RG also inhibited UVB-induced DNA fragmentation (Fig. 6B). Hydrogen peroxide, used as a positive control, induced DNA fragmentation in NHEK.

## Discussion

Protection from UV is important for quality of life. Decrease in oxidative stress from UV irradiation is an effective way to protect skin damage. In this study, we demonstrated that ESG inhibited UVB irradiation-induced oxidative damage and cytotoxicity in cultured skin cells. Treatment with ESG inhibited UVB-induced ROS accumulation in NHEK (Fig. 1) by inducing antioxidant enzymes through the inhibiting the degradation of Nrf2 (Fig. 2–4). Moreover, ESG inhibited UVB-induced apoptosis in NHEK (Fig. 5 and 6). These results indicated that treatment with ESG protected from UVB-induced oxidative stress through inhibiting ROS generation, resulting in attenuating skin cell damage.

In this study, we found ESG and RG, but not ESG residue and OG, decreased UVB-induced ROS accumulation in NHEK (Fig. 1). α-Macrodextrin structure is formed highly branched polymer, α-1,6 linkages, but not α-1,4 linkages.^(21)^ The molecular weight of α-macrodexitrin in natural source glycogen, such as OG, is smaller (<10 kDa) than that in ESG (1,000 to 1,600 kDa).^(16)^ ESG is not completely digested by α-amylase because α-macrodextrin structure in ESG exists in the center of molecule. RG consists of a huge α-macrodextrin polymer.^(15)^ Previously, anti-glycogen monoclonal antibodies, ESG1A9mAb and IV58B6, were developed: ESG1A9mAb mainly recognizes the linear linkages of glycogen,^(16)^ while IV58B6 recognizes α-macrodexitrin structures.^(22)^ This relationship between antibodies and character of glycogen was suggested that α-macrodextrin and the linearity of α-1,4-linkage regions interact with different proteins. This consideration confirmed that RG more strongly suppressed UVB-induced ROS accumulation than ESG in this study. Furthermore, ESG residue, which is completely degraded ESG by α-amylase to glucose and oligosaccharides, did not inhibit UVB-induced ROS accumulation. Thus, α-macrodextrin in ESG is important to inhibit UVB-induced ROS accumulation.

Treatment with ESG increased protein expression level of HO-1, NQO1 and Nrf2 in NHEK, but not mRNA level of Nrf2 (Fig. 2). Inhibitory effect of ESG against UVB-induced ROS accumulation canceled in HO-1- and NQO1-knocked down NHEK (Fig. 4). HO-1, which is an enzyme in heme metabolism, cleaves heme into biliverdin, carbon monoxide and ferrous iron. Biliverdin is converted by biliverdin reductase to bilirubin. Biliverdin and bilirubin has been recognized as the potent antioxidants for scavenging ROS.^(23,24)^ Biliverdin has protected against UVB-induced skin photo-damage in skin.^(25)^ Induction of HO-1 and NQO1, anti-oxidative enzymes, is regulated through the binding of Nrf2 to antioxidant-responsive element.^(26,27)^ ESG increased Nrf2 stability through phosphorylation of Nrf2 at serine residues (Fig. 3A). Kelch-like ECH-associated protein 1 (Keap1), an inhibitor of Nrf2, contributes to the degradation of Nrf2 by ubiquitination, indicating that Keap1 degradation promotes Nrf2 activation and HO-1 and NQO1 expression.^(28)^ Stabilization of the protein level of Nrf2 is regulated by post-transcriptional modification of Nrf2 and Keap1.^(29)^ It has reported that ESG induces NF-κB activity through Toll-like receptor 2 (TLR2) activation.^(30)^ TLRs signaling induces the activation of Nrf2 pathway through autophagy protein-triggered degradation of Keap1.^(31)^ Overexpression of TLR2 upregulates HO-1 expression and decreases oxidative stress.^(32)^ Furthermore, ESG has reported to promote osteogenesis via glucose transporter 1 (GLUT1) and a GLUT1 inhibitor phloretin inhibits ESG-induced phosphorylation of Akt.^(33) ^Akt induces phosphorylation of Nrf2 at Ser40.^(34,35)^ Previously, we have demonstrated that RG induces phosphorylation of Nrf2 at Ser40 through activation of ERK 1/2 and JNK in macrophages.^(17)^ Thus, ESG increased the expression levels of HO-1 and NQO1 through phosphorylation of Nrf2. These results suggest that ESG increases in Nrf2 stability via the activation of TLR2 and GLUT1.

In conclusion, our findings indicate that ESG inhibits UVB-induced ROS accumulation and apoptosis in NHEK. The molecular mechanism of ESG on UVB induced cellular damage is that ESG induces HO-1 and NQO1 expression through increasing stabilization of Nrf2 protein. Thus, ESG might be a new beneficial material for prevention from UVB stress in skin.

## Abbreviations

AMC: 7-amino-4-methyl-coumarin
DAPI: 4',6-diamino-2-phenylindole
DCFH-DA: 2',7'-dichlorodohydrofluorescein diacetate
ESG: enzymatically synthesized glycogen
GLUT1: glucose transporter 1
HO-1: heme oxygenase-1
HRP: horseradish peroxidase
Keap1: Kelch-like ECH-associated protein 1
NF-κB: nuclear factor kappa B
NHEK: normal human epidermal keratinocytes
NQO1: NAD(P)H: quinone oxidoreductase 1
Nrf2: NF-E2-related factor 2
OG: oyster glycogen
RG: resistant glycogen
ROS: reactive oxygen species
TLR: Toll-like receptor
UV: ultraviolet
